# Supplementary material for: Causal Relationship between Aging and Anorexia Nervosa: A White-Matter-Microstructure-Mediated Mendelian Randomization Analysis
Source: Biomedicines. 2024 Aug 16;12(8):1874. doi: 10.3390/biomedicines12081874 (PMC11351342; doi:10.3390/biomedicines12081874)
Supplement: Supplementary file 1 [file biomedicines-12-01874-s001.zip › Supplementary Figure/Figure S8/Leaveoneout plot of MR analysis for TL to ubm-b-1737.pdf]

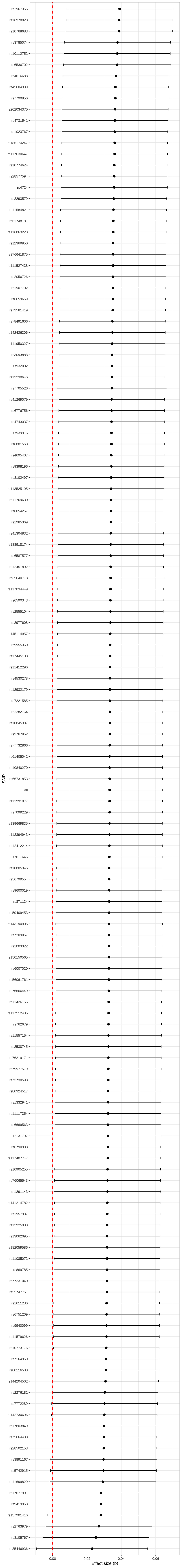

rs2967355
rs16978028
rs10768683
rs3785074
rs10112752
rs6536702
rs4616688
rs45604339
rs7790856
rs202034370
rs4731541
rs1023767
rs185174247
rs117630647
rs10774624
rs28577594
rs4724
rs2293579
rs11584821
rs61748181
rs116863223
rs12369950
rs376641875
rs111527438
rs2056726
rs1907702
rs6659669
rs73581419
rs78491606
rs142426306
rs111950327
rs3093888
rs932002
rs13230646
rs7705526
rs41269079
rs6776756
rs4743037
rs939916
rs6881568
rs4695407
rs9398196
rs8102497
rs113525195
rs11769630
rs6054257
rs1985369
rs41304832
rs188918174
rs6587577
rs12451892
rs35640778
rs117034449
rs6590343
rs2555104
rs2977608
rs145114957
rs9955360
rs17445108
rs11412296
rs4530278
rs12932179
rs7221585
rs2282764
rs10845387
rs3767952
rs77732866
rs61405042
rs10840270
rs66731853
All
rs11991877
rs7099229
rs139669835
rs112394943
rs12412214
rs611646
rs10805346
rs56799554
rs9600019
rs871134
rs59409453
rs143190905
rs7209057
rs1003322
rs150150565
rs6007020
rs56061761
rs76666449
rs11426156
rs117512405
rs762679
rs11557154
rs2538745
rs76219171
rs79977579
rs73730598
rs80324517
rs1332942
rs11117354
rs6669563
rs131797
rs6790988
rs117407747
rs10905255
rs76065543
rs1291143
rs141214782
rs1957937
rs12925933
rs13062095
rs182059586
rs11085072
rs869785
rs77231040
rs55747751
rs1611236
rs6751209
rs6940099
rs11579626
rs10773176
rs7164950
rs80116508
rs144204502
rs2276182
rs7772289
rs142730696
rs17803849
rs75664430
rs28502153
rs3891167
rs5742915
rs11699829
rs17677991
rs9419958
rs137901416
rs2763979
rs8105767
rs35446936
SNP
0.00
0.02
0.04
0.06
Effect size (b)
